# Optimization of convolutional neural network hyperparameters for automatic classification of adult mosquitoes

**DOI:** 10.1371/journal.pone.0234959

**Published:** 2020-07-14

**Authors:** Daniel Motta, Alex Álisson Bandeira Santos, Bruna Aparecida Souza Machado, Otavio Gonçalvez Vicente Ribeiro-Filho, Luis Octavio Arriaga Camargo, Matias Alejandro Valdenegro-Toro, Frank Kirchner, Roberto Badaró

**Affiliations:** 1 University Center SENAI/CIMATEC, National Service of Industrial Learning–SENAI, Computational Modeling and Industrial Technology, Salvador, Bahia, Brazil; 2 University Center SENAI/CIMATEC, SENAI Institute of Innovation (ISI) in Health Advanced Systems (CIMATEC ISI SAS), Salvador, Bahia, Brazil; 3 University Center SENAI/CIMATEC, High Performance Computing Center–SENAI CIMATEC, Salvador, Bahia, Brazil; 4 German Research Center for Artificial Intelligence (DFKI), Bremen, Germany; Newcastle University, UNITED KINGDOM

## Abstract

The economic and social impacts due to diseases transmitted by mosquitoes in the latest years have been significant. Currently, no specific treatment or commercial vaccine exists for the control and prevention of arboviruses, thereby making entomological characterization fundamental in combating diseases such as dengue, chikungunya, and Zika. The morphological identification of mosquitos includes a visual exam of the samples. It is time consuming and requires adequately trained professionals. Accordingly, the development of a new automated method for realizing mosquito-perception and -classification is becoming increasingly essential. Therefore, in this study, a computational model based on a convolutional neural network (CNN) was developed to extract features from the images of mosquitoes and then classify the species *Aedes aegypti*, *Aedes albopictus*, and *Culex quinquefasciatus*. In addition, the model was trained to detect the mosquitoes of the genus *Aedes*. To train CNNs to perform the automatic morphological classification of mosquitoes, a dataset, which included 7,561 images of the target mosquitoes and 1,187 images of other insects, was acquired. Various neural networks, such as Xception and DenseNet, were used for developing the automatic-classification model based on images. A structured optimization process of random search and grid search was developed to select the hyperparameters set and increase the accuracy of the model. In addition, strategies to eliminate overfitting were implemented to increase the generalization of the model. The optimized model, during the test phase, obtained the balanced accuracy (BA) of 93.5% in classifying the target mosquitoes and other insects and the BA of 97.3% in detecting the mosquitoes of the genus *Aedes* in comparison to *Culex*. The results provide fundamental information for performing the automatic morphological classification of mosquito species. Using a CNN-embedded entomological tool is a valuable and accessible resource for health workers and non-taxonomists for identifying insects that can transmit infectious diseases.

## Introduction

The economic impact of vector-borne diseases is significant. For the governments in endemic countries, the economic impact includes the costs spent in vector control and case-management activities [1–3]. In addition, the global economic cost spent to combat these illnesses is measured in billions of dollars annually, and it also includes other types of costs such as loss of work and school days [4–7]. Many arboviruses cases are not reported, making it difficult to estimate the true economic impact of the diseases [8].

Dengue, chikungunya, and Zika are the most common viral diseases transmitted by mosquito-vectors [9–11] and negatively impact the public health and result in economic damage worldwide [12,13]. The effective prevention and control of arboviruses depends on timely and accurate detection of elevated viral activity in the population [14].

According to the World Health Organization (WHO), the global incidence of dengue has dramatically grown in the recent decades and approximately half the world's population is at risk. The number of dengue cases reported to WHO increased approximately 6 fold, from <0.5 million in 2010 to over 3.34 million in 2016. Notably, a substantial number of cases in the Americas were reported in Brazil. Globally, there are an estimated 390 million dengue infections each year [15]. The global cost of dengue was U$ 8.9 billion in 2013, and in 34% of the cases, the patients were not medicated [8]. The control and reduction of dengue cases can globally save billions of dollars.

Regarding the Zika virus, the United Nations Development Program, in partnership with the Red Cross International Federation, evaluated the socio–economic impacts of the Zika virus in Latin American and Caribbean countries, especially in Brazil, Colombia, and Suriname [16]. Their report asserted that Zika was responsible for tangible losses in the gross domestic product, estimated between seven and eight billion dollars in the period from 2015 to 2017, thereby imposing an immediate burden on health systems and social assistance.

The economic impact goes beyond the costs related to public health and the reduction in the gross domestic product of the countries. It directly affects the household economy. In the state of Orissa, in India, 10% of the monthly family income was spent on health-care expenses for treating the chikungunya effects. Among the people interviewed, on average, the workers lost 35 days of work because of the illness [17].

The mosquito of the species *Aedes aegypti* is the main transmission vector of arboviruses and infects millions of people worldwide [18,19]. In addition, the species *Aedes albopictus* is another transmitter vector of these diseases, and in the last three decades, its population has exploded geographically worldwide [20–23]. *Culex quinquefasciatus*, other than being a disease-transmitting vector, is, along with *Ae*. *aegypti*, one of the most common urban mosquitoes in tropical and subtropical environments, causing discomfort to humans [24].

The proximity of mosquito-vector breeding sites to human habitation poses a significant risk factor due to the diseases that these species transmit [25,26]. Currently, no specific treatment or commercial vaccine exists for the control and prevention of arboviruses; therefore, the population control of mosquitoes is the only preventive measure [27–29]. Entomological characterization is critical to acquiring the information on mosquito behavior; however, the current practice that is used to identify insect species is manual, time consuming, and requires experienced professionals [30–32]. Accordingly, the development of a new automated method for performing mosquito perception and classification is becoming increasingly essential [31,33,34]. Despite being a problem in different parts of the world, especially in tropical countries, vector control and prevention programs are the only strategies in the hands of governments to reduce the incidence of arboviruses [7,35].

Recently, new models based on machine learning and deep learning have been developed to automatically classify and detect the species of mosquitoes [36–39]. However, as discussed by Batista et al., the efficiency of these tools depends on the knowledge of the temporal space of the transmitting vectors [40]. The time spent between the capture of the sample and the analysis thereof in a laboratory affects this efficiency.

In a recent study, we demonstrated that CNNs can be used by health workers and non-taxonomists to autonomously classify mosquitoes to aid in the screening of possible vectors of arboviruses [41]. The aforementioned study presented important results using LeNet [42], AlexNet [43], and GoogLeNet [44] neural networks. However, it also recommended that more complex networks must be used to improve the model accuracy, and that significant amount of data were required to increase the reliability of any autonomous method to recognize objects. Both the recommendations are addressed in this study.

In addition to using more complex architectures, deep-learning algorithms require optimization in different contexts and are the most difficult to be optimized among all the neural networks [45]. CNNs demand the definition of several training parameters that are not automatically adjusted during the learning process. It is fairly common to invest months to optimize a limited number of training parameters, and, accordingly, many optimization techniques have been developed [45]. Notably, random-search and grid-search techniques are the most frequently used strategies to optimize the hyperparameters [46].

Because the prevention and control strategies to counter arboviruses have not shown satisfactory results in reducing disease transmission, utilizing automated and efficient tools of classifying mosquitoes can be significantly useful to improve the programs to control these diseases. Accordingly, the objective of this study is to develop a CNN-based entomological model for field use by experts and non-specialists to automate the classification of *Ae*. *aegypti*, *Ae*. *albopictus*, and *C*. *quinquefasciatus*, as well as allow the detection of the genus *Aedes*. More complex state-of-the-art CNN architectures, such as Residual Network (ResNet) [47], VGG [48,49], InceptionV3 [50], Xception [51], and DenseNet [52], were used in the model, and the hyperparameters were optimized using a random- and grid-search approaches to increase the model accuracy. We must highlight that this study meets the current guidelines recommended by WHO. The prevention and control of arboviruses depends on the effectiveness of vector-control measures. Sustained community involvement can substantially improve the vector-control efforts.

## Materials and methods

### Sample collection and ethics statement

No permits were required for sampling for this study, as the field sampling did not involve any endangered or protected species.

The mosquito samples used for image capture were obtained from the same database as used in the study by Motta et al. [2019] [41] with several adaptations (i.e., inclusion of new samples). the newly included samples were kindly donated by the Parasitology Laboratory of the Federal University of Bahia–UFBA (Salvador, Brazil) and Oswaldo Cruz entomology Institute–FIOCRUZ (Rio de Janeiro, Brazil). Some of the new samples were also directly collected from the field by a trained entomologist and added to the SENAI CIMATEC Biotechnology Laboratory database (see Table 1).

**Table 1:** Identification of mosquito samples used in this study to obtain the images and database.

Sample	Sample origin	Number	Description
*Ae*. *Aegypti*	UFBA	16	10 females and 6 males
*Ae*. *Aegypti*	FIOCRUZ	03	Females
*Ae*. *Aegypti*	Field (database CIMATEC)	120	94 females and 26 males
*Ae*. *Albopictus*	UFBA	10	5 females and 5 males
*Ae*. *Albopictus*	FIOCRUZ	03	Females
*Ae*. *Albopictus*	Field (database CIMATEC)	94	71 females and 23 males
*C*. *quinquefasciatus*	FIOCRUZ	03	Females
*C*. *quinquefasciatus*	Field (database CIMATEC)	110	97 females and 13 males

In this study, 359 mosquito samples were used. Of these samples, 139 were the specimens of *Ae*. *aegypti*, 107 of *Ae*. *albopictus*, and 113 of *C*. *quinquefasciatus*. Field sampling was performed between September and October 2017 and between March and April 2018 in the city of Salvador in two collection areas (Bahia, Brazil). CDC light traps (Centers for Disease Control and Prevention miniature light traps) and suction tubes were used for collecting adult insects. The captured specimens were euthanized using ethyl acetate and stored in entomological collection tubes until identification was performed by an entomologist.

### Image acquisition

In this study, we utilized a supervised deep learning approach. Therefore, the structuring and labeling of the dataset is a fundamental step for performing the correct training and evaluation of the CNN.

Of the 7,561 images of the target mosquitoes, namely, *Ae*. *aegypti*, *Ae*. *albopictus*, and *C*. *quinquefasciatus* used in this study, 95% (7,180) were directed acquired from the samples collected, and the remaining 5% (381) were donated by the Institute of Entomology Oswaldo Cruz of FIOCRUZ (Rio de Janeiro, Brazil) and also extracted from the ImageNet database. The species were photographed using various cameras, such as a Leica DMC2900 (Leica Microsystems, Heerbrugg, Switzerland) coupled to a stereoscopic Leica M205C at the Oswaldo Cruz Institute of Entomology at FIOCRUZ (Rio de Janeiro–Brazil), Canon Power Shot D30 (Canon, Tokyo, Japan) coupled to a Wild M3C stereomicroscope (Leica Microsystems, Heerbrugg, Switzerland) at SENAI CIMATEC (Salvador–Brazil), and mobile-phone cameras (Samsung J5, Seoul, South Korea and Apple iPhone 7, Cupertino, California, USA) [41].

**Fig 1:**
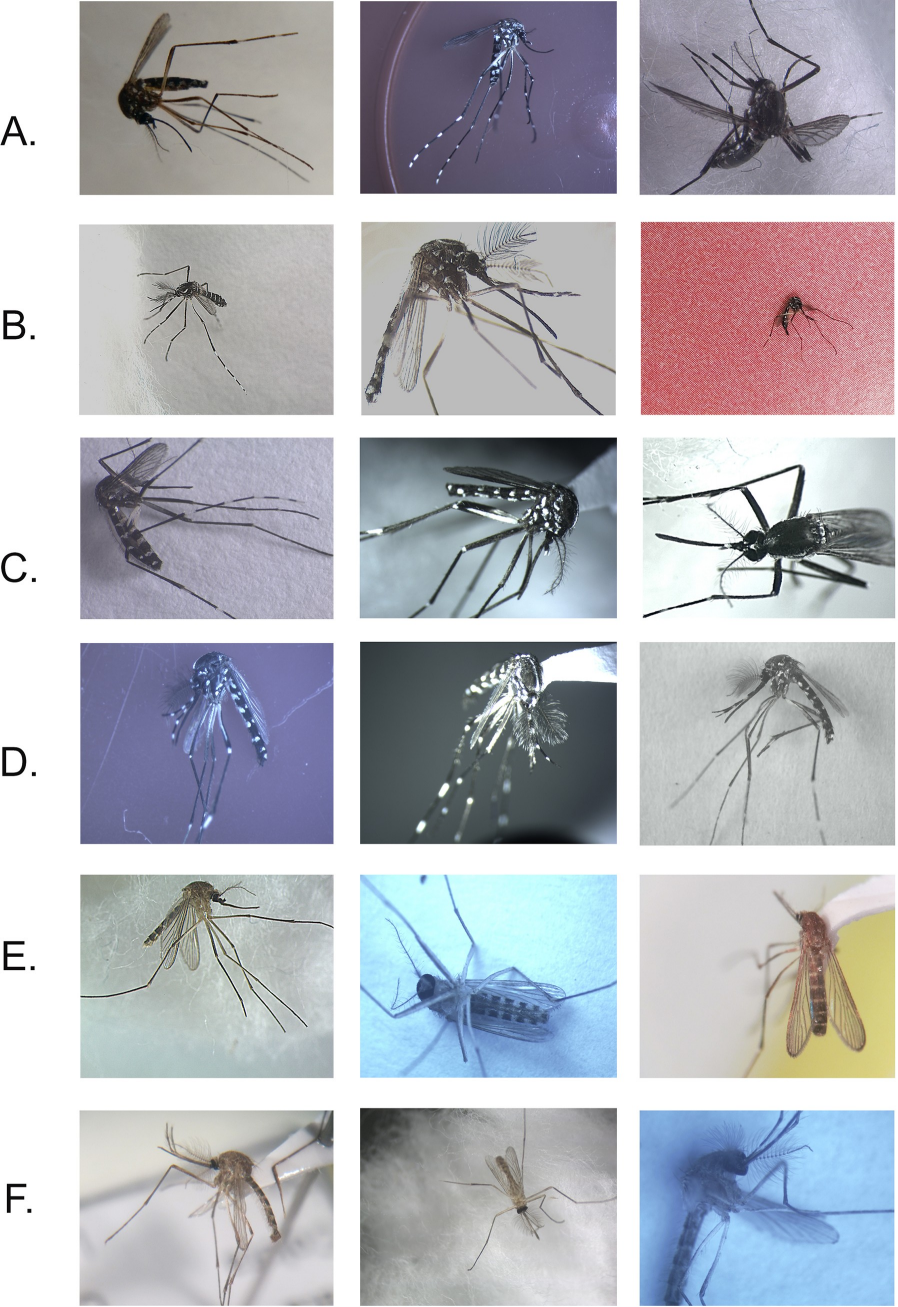
Sample of the images used in developing the model. (A) *Ae*. *aegypti* female. (B) *Ae*. *aegypti* male. (C) *Ae*. *albopictus* female. (D) *Ae*. *albopictus* male. (E) *C*. *quinquefasciatus* female. (F) *C*. *quinquefasciatus* male.

The images were collected at different resolutions and different angles of the mosquito in the image. The objective was to develop a classification model that could be generalized. In Fig 1, we depict a sample of the images used in this study.

All the images used in this study were evaluated by an entomologist to validate their labeling.

In addition, 1,187 images of spiders, beetles, and bees were acquired from the Internet and labeled as “others.” This was done to train the model and prevent another species of insects being labeled as one of the target classes. All the images used in this study were analyzed and classified by an entomologist. In Table 2, we list the number of images by class used in this study.

**Table 2:** Number of images by class.

Class/genre	Number of images
*Ae*. *aegypti* female	1,193
*Ae*. *aegypti* male	1,562
*Ae*. *albopictus* female	1,448
*Ae*. *albopictus* male	1,360
*C*. *quinquefasciatus* female	1,025
*C*. *quinquefasciatus* male	973
Others	1,187
**TOTAL**	**8,748**

### Dataset split in training, validation, and testing

In this study, the method used to develop the automatic-classification model was based on the one proposed by Motta et al. [41].

The dataset was divided to be used in the following three distinct phases: training, validation, and testing. First, in the training phase, the dataset with its labels is presented to the algorithm, and the model is trained using the attributes of each class. Second, in the validation phase, which is simultaneously performed with the training phase, the objective is to measure the quality of the training with new data. In this phase, the classification is evaluated and network weights are adjusted with the objective of increasing the accuracy and reducing the loss. Third, in the testing phase, a new dataset is presented to the model. No weight adjustments are performed at this stage, and the target is to evaluate the generalization ability of the model.

The split into the three phases was randomly adopted as follows: 60% for training, 20% for validation, and 20% for testing. The images were randomly divided in each phase according to the defined percentages.

### Performance-evaluation metrics

During the training and validation phases, the metrics used to evaluate the performance of the model were loss function and accuracy. The loss function used in this study was the cross-entropy cost function.

In the testing phase, the network performance was evaluated using a confusion matrix. Notably, the dataset used in this study was unbalanced; i.e., each class had different number os datapoints in the database and, therefore, a different weight in the overall result. Therefore, in this study, we used a metric that considered the effect of this imbalance. Although several metrics are currently used from the confusion matrix, balanced accuracy (BA) represents the overall performance when the database is unbalanced [53]. In Table 3, we present a 2 x 2 confusion matrix. Eqs 1, 2, 3 and 4 present the calculation methods of the metrics of this matrix.

$$Precision:\;PR=\frac{TP}{TP+FP}$$ (1)

$$True–Positive\;Rate:\;TPR=\frac{TP}{TP+FN}$$ (2)

$$True–Negative\;Rate:\;TNR=\frac{TN}{TN+FP}$$ (3)

BalancedAccuracy:BA=0,5×(TPR+TNR) (4)

**Table 3:** Confusion matrix 2 x 2 metrics.

		Predicted Class	
		Positive	Negative
Actual Class	Positive	**TP**	**FN**
	Negative	**FP**	**TN**

In this study, additionally, the classification process also considered matrices above of two classes. The way indicators are calculated in these cases changes. To obtain a single performance-evaluation metric, we first used the performance analysis for each class, according to the equations presented in Table 4.

**Table 4:** Confusion matrices of the above-mentioned two classes.

		Predicted Class			TPR	TNR	BA
		Class 1	Class 2	Class 3			
Actual Class	Class 1	***a***	***b***	***c***	$\frac{a}{a+b+c}$	$\frac{e+f+h+i}{d+e+f+g+h+i}$	${BA}^{1}=\frac{TVP+TVN}{2}$
	Class 2	***d***	***e***	***f***	$\frac{e}{d+e+f}$	$\frac{a+c+g+i}{a+b+c+g+h+i}$	${BA}^{2}=\frac{TVP+TVN}{2}$
	Class 3	***g***	***h***	***i***	$\frac{i}{g+h+i}$	$\frac{a+b+d+e}{a+b+d+e+g+h}$	${BA}^{3}=\frac{TVP+TVN}{2}$
Precision		$\frac{a}{a+d+g}$	$\frac{e}{b+e+h}$	$\frac{i}{c+f+i}$			$\frac{{BA}^{1}+{BA}^{2}{+BA}^{3}}{3}$

For the cases of the above-mentioned two classes, the overall accuracy result of the model was considered the mean of the balanced accuracies for each class.

### Data augmentation

When a CNN is used for the visual recognition of objects, with low data availability, data augmentation is commonly used [54]. In this study, the training and validation datasets were computationally augmented from the original images obtained. The test dataset was not augmented.

Various data-augmentation parameters (rotation, width-shift range, height-shift range, brightness, and zoom) were randomly tested, and after a performance evaluation, a grid search followed by two stages was developed.

In the first stage, the objective was to identify the best set of hyperparameters for generating new images, as presented in Table 5. Initially, three hyperparameters were tested: rotation, width-shift range, and height-shift range. This definition was supported by the results obtained from the random search performed previously. Subsequently, the variation values of each hyperparameter was determined. For rotation, two levels were tested (45° and 90°), and for width- and height-shift ranges, three levels (5%, 15%, and 25%) were tested. Finally, the network underwent the training and validation phases for seven different combinations, including the comparison without data augmentation. On the basis of the results, the set of hyperparameters with better performances in terms of accuracy and cross-entropy loss, both in the validation phase, was defined as the standard to be used in this study.

**Table 5:** Grid search–hyperparameters for dataset augmentation.

Data-Augmentation Hyperparameters	Experiments						
	1°	2°	3°	4°	5°	6°	7°
Rotation	No augmentation	45°	45°	45°	90°	90°	90°
Width-shift range		5%	15%	25%	5%	15%	25%
Height-shift range		5%	15%	25%	5%	15%	25%

In the second stage, the objective was to evaluate the number of times the data should be magnified. Using the set selected in the first stage, the dataset was multiplied by 5, 10, 15, and 20. The multiplier with the best performance, considering the same criteria as those in the previous stage, was then selected for the developing the optimized model.

### Definition of the CNN architecture and optimization of hyperparameters for the classification layers

In this study, we used pre-trained CNN models available in the Keras library [55]. Using a pre-trained model, also known as transfer learning, is important to improve the performance of image classification, as its weights have previously been optimized with attributes that are important in most computer-vision problems. The use of the transfer-learning technique was possible in this study, as the dataset used had images that were similar to those used in ImageNet. If the images were not similar to those used to train the models available, it would have been necessary to retrain some layers.

The CNN selection and optimization of the hyperparameters was performed in five steps. In the first step, the target was to randomly evaluate the performance of different network architectures and hyperparameters in the process of automatic classification of mosquitoes, in terms of accuracy and loss in the training and validation phases.

In the second step, 16 experiments were developed, as listed in Table 6. Considering the performance achieved by Xception and DenseNet201 in the first step, those architectures were selected as the ones to be used for developing the model.

**Table 6:** Grid search—CNN architecture and hyperparameters for classification layers.

CNN e Hyper.	Experiments															
	1°	2°	3°	4°	5°	6°	7°	8°	9°	10°	11°	12°	13°	14°	15°	16°
CNN	Xcp	Xcp	Xcp	Xcp	Xcp	Den	Den	Den	Den	Den	Den	Den	Den	Den	Den	Den
Epochs	200	200	200	200	300	200	200	200	200	200	200	200	200	200	200	300
LR	4e-7	4e-6	2e-5	1e-4	4e-6	4e-6	1e-5	2e-5	3e-5	6e-5	1e-4	2e-4	3e-4	6e-4	1e-3	4E-6

(Epochs: number of epochs; LR: initial learning rate; Xcp: Xception network; Den: DenseNet201 network).

On the basis of the results of the second step, the five best-performing models in terms of accuracy in the validation phase and the five best ones in terms of loss function in the validation phase were selected for classifying the test dataset.

Notably, the values of the initial weights assigned in a neural network training begin with random values. As the learning process evolves, these weights are adjusted. However, the initial randomness of these values may affect the final performance result. Accordingly, the fourth step was to evaluate this variation, and the two experiments with the best overall accuracy performances were retrained two more times, following which their new models were resubmitted to the test dataset evaluation.

In the last step, the batch size for the best set obtained in the fourth step varied as 32, 64, 128 and 256.

### Evaluation of the optimized model

To evaluate the applicability of the model in the community (i.e., among non-specialists), the classification of the species may not be as important as the detection of the genus *Aedes*. Accordingly, for the community and public programs regarding the prevention of arboviruses, the detection of the mosquito *Aedes* is considered an important factor. Another important point to evaluate the model is its ability to recognize other insects and distinguish them from the target mosquitoes of this study.

Accordingly, the optimized model was tested in the following three distinct scenarios: (a) six classes—in this case, the classification of the three species, as well as the differentiation between males and females, is evaluated; (b) two classes—in this case, there is only the differentiation between *Aedes* and *Culex*; (c) seven classes—in this case, a another insect category is included to evaluate the behavior of the network when an image of a non-mosquito is presented.

### Angle of the mosquito in the image

**Fig 2:**
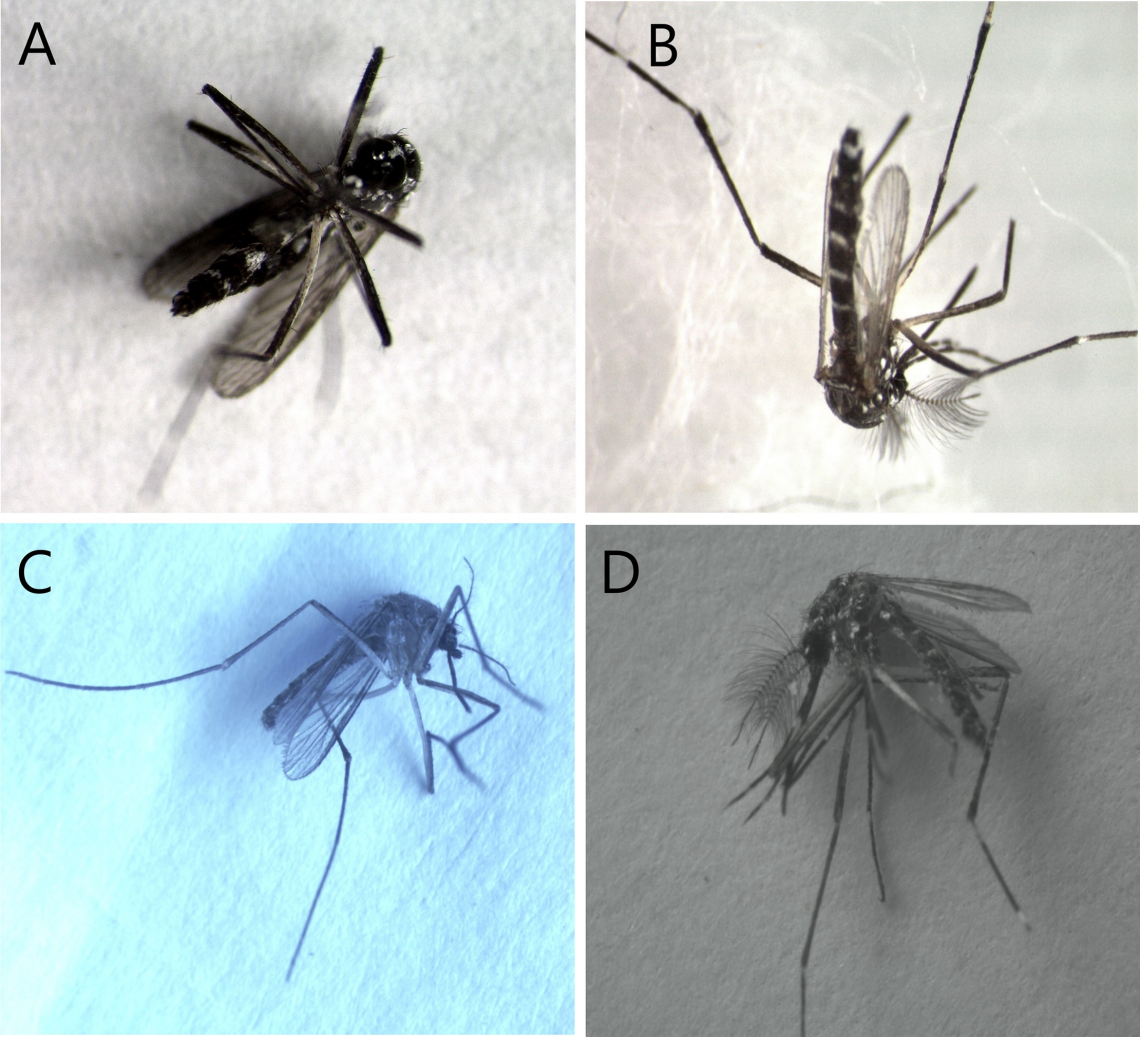
Samples of images at different angles of mosquitoes. (A) Bottom angle. (B) Back angle. (C) Right angle. (D) Left angle.

Another evaluation performed in this study was on the effect of the object angle in the image on the prediction performance of the model. A photography expert separated the test database from the six classes in the following four different sets: (A) frontal and bottom angles, which consider the photos taken from the front of the head region and below the thorax of the mosquito, respectively; (B) back angle, which comprises the region of the wings; (C) right angle, and (D) left angle. In Fig 2, we depict a sample from each of the four sets.

The test dataset had 344 images with front and bottom angles, 287 images with back angle, 460 with right angle, and 419 with left angle.

## Experimental setup

**Fig 3:**
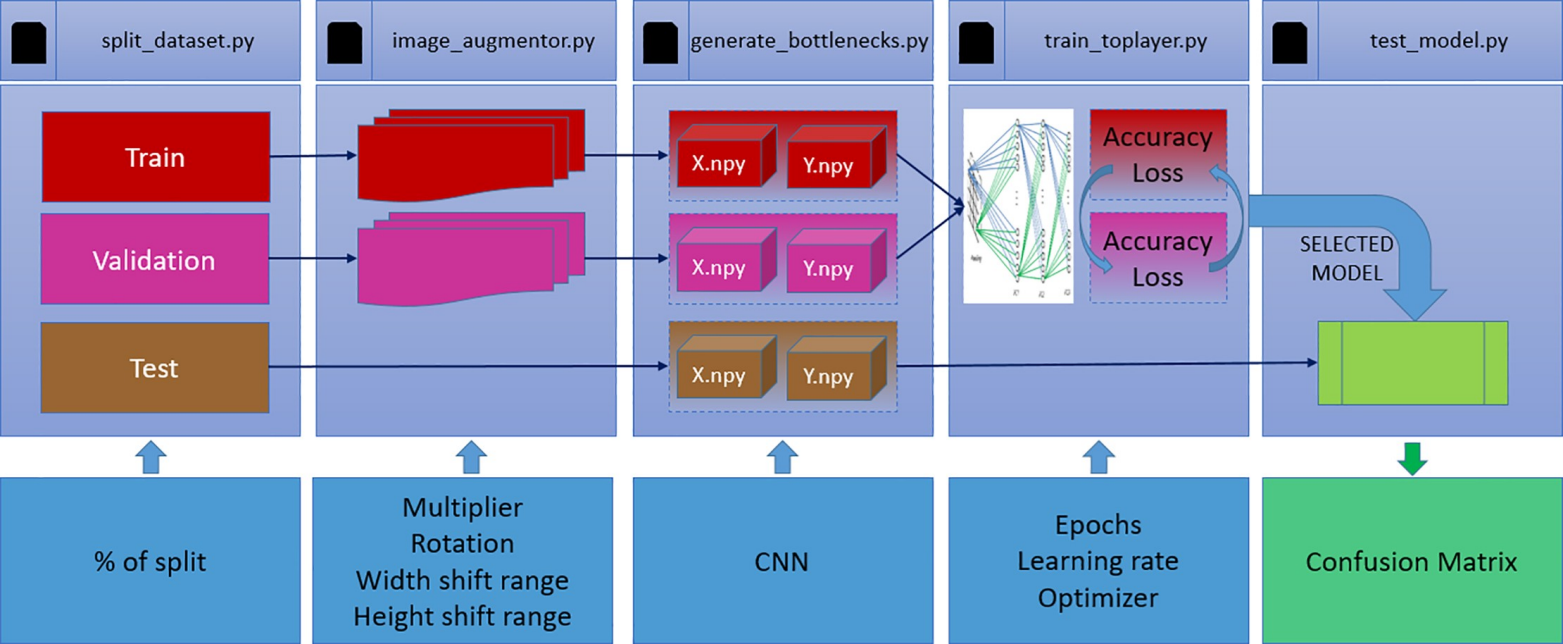
Programs used for the development of work.

The experiments were performed using Python as the programing language and Keras framework for training the models. The workflow adopted is depicted in Fig 3 and can be divided into the following 3 steps: data pre-processing, transfer learning, and model training and testing. As previously mentioned, for every model trained, the dataset was split into training, validation, and testing phases, with the proportions of 60%, 20%, and 20% respectively.

### Data pre-processing

In this step, the dataset was computationally augmented with random variations in rotation, height, and width. Only the training and validation images were augmented. Generating new images took an average of 5 h for the training dataset and 2 h for the validation dataset, considering the augmentation of the images for the six classes in 20 times.

### Transfer learning

In this step we aimed to extract the attributes of the images by using some of the pre-trained networks. For each of the three phases of the model development (training, validation, and testing), two vectors *X* and *Y* were generated, where *X* denotes the attribute vector of the images that was used to characterize the output signal present in vector *Y* according to its label, and *Y* denotes the label vector.

The main purpose of CNN convolutional layers is to capture the relevant attributes that characterize and generalize the classification process. The input signal (*x*) requires the specification of the attributes of that have the most important role in its prediction [56]. During the training epochs, significant time is spent in the process of compressing the input data for realizing an efficient representation of the output data. It is a process to identify the most relevant information for the identification process [57].

The CNN architecture is selected in this stage. The program was developed to enable the use of any of the pre-trained models that are currently available in the Keras library (https://keras.io/applications/): Xception, VGG16, VGG19, ResNet50, InceptionV3, InceptionResNetV2, MobileNet, DenseNet, NASNet, and MobiliNetV2.

In this study, the average generation time of the attribute vectors in the training phase was 30 h, in the validation phase was 2.5 h, and in the test phase was 10 min. This time represents the average of each time the program is used while generating attribute vectors for the six classes.

### Model training and testing

This step represents the classification layers. As explained earlier, it is at this stage that 2D feature maps are converted to a 1D feature vectors and object classification or recognition is performed. In addition to using the attributes vectors generated using the previous program, in this step, we select the values of the hyperparameters, such as number of epochs, initial learning rate, and optimizer, that directly affect the learning process of the network.

The callbacks dropout and reduce learning rate on plateau functions are used for reducing the risk of overfitting and improving the performance of the classification process.

By running this program, the model after undergoing training and validation phases is saved. This is important to enable the use of the model with the test dataset and, thus, evaluate the performance of the network in classifying new images. In this study, the mean training time of the network, for 200 epochs, was 2 h.

The model saved with the weights trained after using all the previous programs is evaluated in the test phase. At this stage, a new dataset is presented, and a confusion matrix is generated, enabling the evaluation of the generalization capability for each class.

## Results and discussion

### Random search

The setup of the grid-search plan to optimize the data-augmentation parameters and model hyperparameters was preceded by a random search. This search was conducted to support the definition of the hyperparameters that were varied and the scope of this variation. The dataset with six classes was the one used for the model optimization. In Table 7, we present the results obtained after the last epoch of the network for the 24 experiments performed during the random search.

**Table 7:** Random search results.

#	M	CNN	Epochs	Optimizer	LR	Results			
						L_t	Acc_t (%)	L_v	Acc_v (%)
1	10*	ResNet50	200	SGD	1e-3	1.77	21.0	1.77	21.0
2	0	ResNet50	200	SGD	1e-3	1.65	28.8	1.64	28.3
3	0	ResNet50	200	RMSprop	1e-3	1.48	39.3	1.50	36.8
4	0	ResNet50	200	Adagrad	1e-3	1.54	36.6	1.55	35.7
5	0	Xception	200	RMSprop	1e-3	0.06	98.3	1.41	79.9
6	0	VGG16	200	RMSprop	1e-3	0.42	83.4	0.81	75.1
7	0	DenseNet201	200	RMSprop	1e-3	0.01	99.6	1.01	87.2
8	0	DenseNet201	120	RMSprop	1e-3	0.02	99.4	0.96	87.2
9	2*	VGG16	200	RMSprop	1e-3	1.69	26.1	1.77	24.7
10	0	VGG16	300	RMSprop	1e-3	0.38	86.1	0.86	75.1
11	0	VGG16	200	RMSprop	1e-2	1.77	21.3	1.77	21.3
12	0	VGG16	200	RMSprop	1e-4	0.62	77.6	0.80	71.1
13	0	InceptionV3	200	RMSprop	1e-3	0.06	97.6	1.40	79.7
14	0	Xception	200	Adam	1e-3	0.04	98.8	1.34	80.3
15	0	Xception	200	Adam	1e-4	0.11	97.1	0.72	79.8
16	0	Xception	200	Adam	1e-5	0.48	82.8	0.63	76.0
17	2	Xception	200	Adam	5e-5	0.24	91.7	0.65	78.4
18	2	DenseNet201	200	Adam	5e-5	0.24	91.3	0.62	79.2
19	2	DenseNet201	200	Adam	1e-5	0.44	83.6	0.61	77.6
20	2	DenseNet201	200	Adam	2e-5	0.33	87.8	0.59	78.8
21	2	VGG16	200	Adam	1e-3	0.54	78.8	0.99	66.5
22	2	VGG16	200	Adam	1e-4	0.76	71.1	0.95	63.1
23	10	DenseNet201	200	Adam	2e-5	0.21	92.3	0.42	85.9
24	15	DenseNet201	200	Adam	2e-5	0.19	92.9	0.41	86.9

M: Data-augmentation multiplier; LR: Learning rate; L_t: Training loss; Acc_t: Training accuracy; L_v: Validation loss; Acc_v: Validation accuracy.

* Dataset augmentation with indiscriminate variation in hyperparameters.

**Fig 4:**
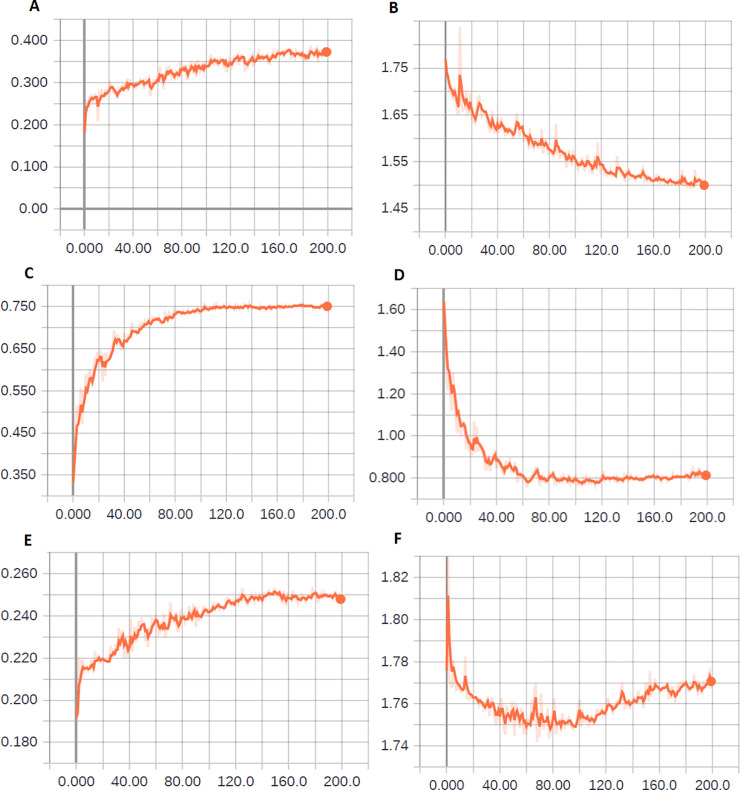
Evolution of network learning upon using ResNet50 and VGG16. (A) Accuracy in the validation phase–experiment 3 (ResNet50). (B) Cross entropy in the validation phase–experiment 3 (ResNet50). (C) Accuracy in the validation phase–experiment 6 (VGG16). (D) Cross entropy in the validation phase–experiment 6 (VGG16). (E) Accuracy in the validation phase–experiment 9 (VGG16). (F) Cross entropy in the validation phase–experiment 9 (VGG16).

**Fig 5:**
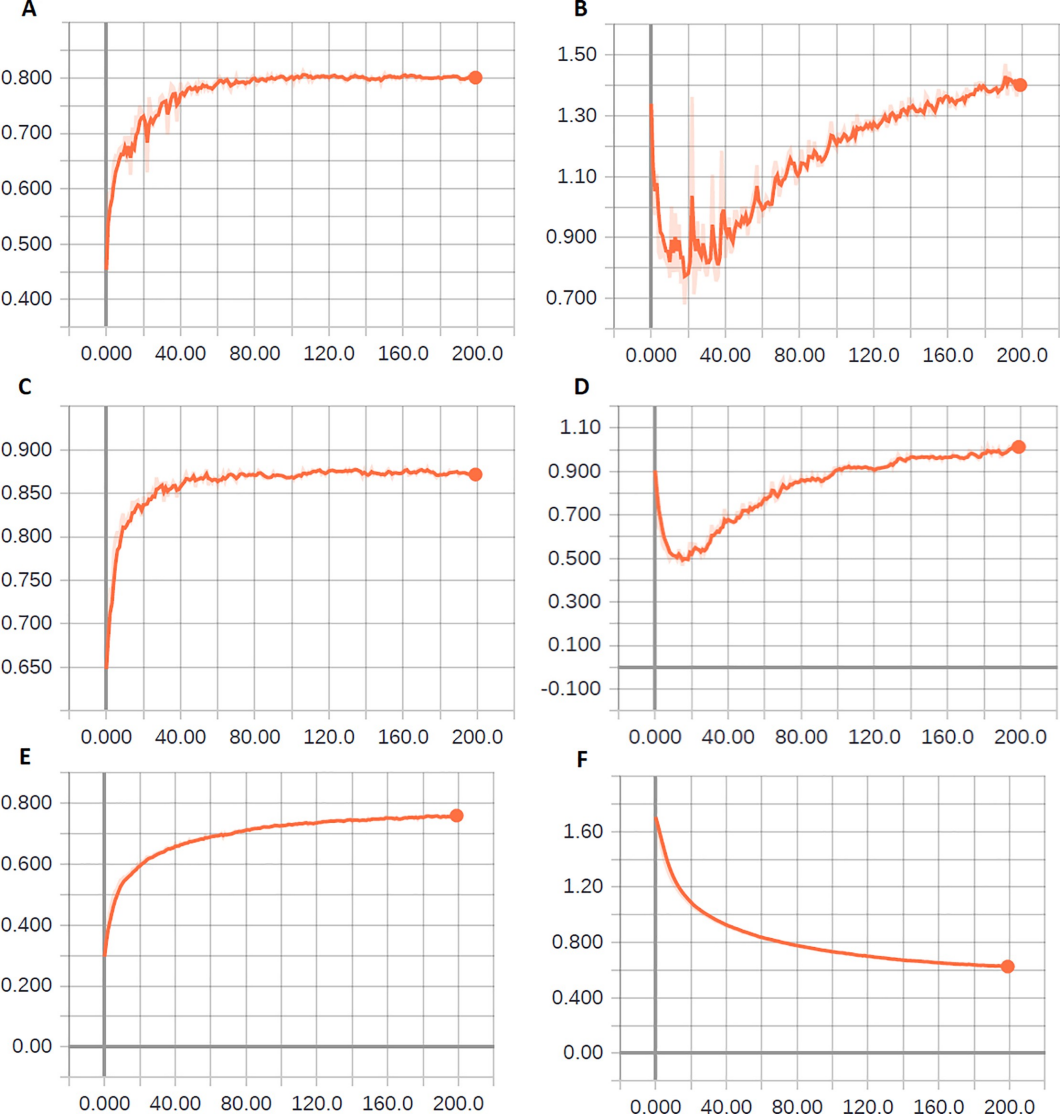
Evolution of network learning by using Xception and DenseNet201. (A) Accuracy in the validation phase–experiment 5 (Xception). (B) Cross entropy in the validation phase–experiment 5 (Xception). (C) Accuracy in the validation phase–experiment 7 (DenseNet201). (D) Cross entropy in the validation phase—experiment 7 (DenseNet201). (E) Accuracy in the validation phase after reducing the initial learning rate to 1E-5 –experiment 16 (Xception). (F) Cross entropy in the validation phase after reducing of the initial learning rate to 1E-5 –experiment 16 (Xception).

**Fig 6:**
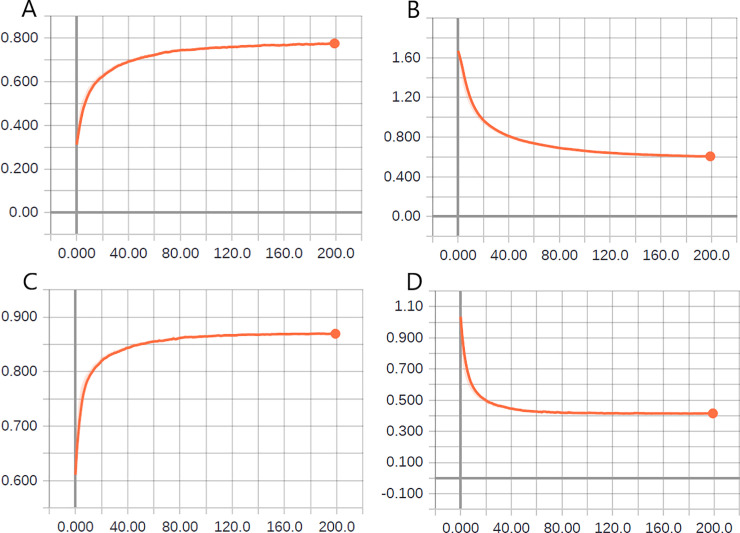
Evolution of the learning of the DenseNet network after dataset augmentation. (A) Accuracy in the validation phase—experiment 20. (B) Cross entropy in the validation phase—experiment 20. (C) Accuracy in the validation phase—experiment 24. (D) Cross entropy in the validation phase—experiment 24.

For correctly interpreting the results, we must analyze the learning curves of each model. In Figs 4, 5 and 6, we depict some curves of learning process of the experiments and exemplify the criteria used for developing the grid-search plan. In Fig 4, we depict the cross-entropy (loss function) and accuracy in the validation phase with the use of ResNet50 and VGG16 (see Table 7).

ResNet was the winner of the ILSVRC-2015 challenge in the category of object classification with a margin of error-top-5 of 3.57% [47,58,59]. However, in the classification of adult mosquitoes with the parameters used in this study, it did not perform satisfactorily. It should be noted that no optimization was performed for using ResNet in this study. Because of its performances in experiments 2, 3, and 4, using different optimizers (SGD, RMSprop, and Adagrad, respectively), it was decided not to include the ResNet architecture in the grid search. Notably, in both Fig 4A and 4B, underfitting is indicated. That is, with a greater number of epochs, this network tends to improve its performance.

Another important evaluation for the random-search phase was related to the effect of the dataset augmentation. The benefits of dataset augmentation were demonstrated in a recently performed work [60]. By carefully evaluating the images generated in experiment 9, we verified that there were no mosquitoes in them, which was defined by reviewing the number of hyperparameters and their amplitudes for generating new images for the dataset. The augmentation of the images from experiment 17 follows a new configuration. The new results confirm that dataset augmentation can improve the model performance in the classification of images of adult mosquitoes.

In Fig 5, we depict the cross entropy and accuracy in the validation phase using the Xception and DenseNet201 networks. From Fig 5B and 5D, it is evident that upon reducing the number of epochs, the minimum cross-entropy value is achieved, and that at this point (i.e., approximately 20 epochs), the error in the validation phase begins to increase. However, the validation accuracy of the model still increases along the number of epochs.

Accuracy and loss might be inversely correlated to each other; i.e., the higher the accuracy, the lower the loss. However, during the learning process of a neural network, the loss function can increase, despite increased or maintained accuracy. We must correctly understand this phenomenon to decide which is the "best" set of hyperparameters for a given application.

The loss function measures the distance between the actual and predicted values. The predicted value is probabilistic and the real value deterministic. Considering a hypothetical situation of a real image of an *Ae*. *aegypti* male mosquito, the actual value assumes that the image is 100% likely to be of an *Ae*. *aegypti* male mosquito. However, upon presenting this image to the prediction algorithm, this evaluation can vary between 0% and 100%. The loss function measures this variation between the actual and predicted values.

Accuracy measures the predicted value as deterministic. When evaluating the same above-mentioned image, if the algorithm classifies the image as being 80% likely to be of an *Ae*. *aegypti* male mosquito and 20% likely to be of an *Ae*. *albopictus* female, for the value of accuracy, the model would have 100% accuracy.

Therefore, if at a given epoch of the network training, the model predicts that an image is 80% likely to be of an *Ae*. *aegypti* male mosquito and in the subsequent epoch, the same model predicts that the same image is 60% likely to be of an *Ae*. *aegypti* male mosquito, then the loss function increases, but the accuracy remains the same.

This effect should not be confused with overfitting. Overfitting is related to the inability to generalize the model when new data, which are not used in training or validation phase, are presented [45]. To detect overfitting, we must evaluate the image-classification performance when using the test dataset.

Despite the importance of the loss function, in this study, the BA in the test phase is considered more relevant.

Alternatively, we can minimize this effect is by changing the initial learning rate. The performance of a deep-learning-based method significantly depends on the selection and evolution of the learning rate and can considerably increase the model of the training process [61]. In Fig 5E and 5F, we depict the effect of reducing the initial learning rate while using Xception.

However, reducing the initial learning rate alters the learning speed of the network and consequently, its final performance. Therefore, we investigated the effects of the initial learning rate at the grid-search stage. Fig 5 depicts underfitting, reinforcing the importance of assessing the number of epochs in addition to the initial learning rate.

In Fig 6, we depict the cross entropy and accuracy in the validation phase of experiments 20 and 24 (see Table 7) obtained using DenseNet201. In addition to the fact that the dataset augmentation improves the performance of the network in the classification process, we verified that it accelerates the learning process as well.

### Optimization of hyperparameters for data augmentation

The data-augmentation hyperparameters were optimized in two stages. In the first stage, the best set of hyperparameters was defined for generating new images. In the second stage, we evaluated the number of times the data should be augmented.

**Fig 7:**
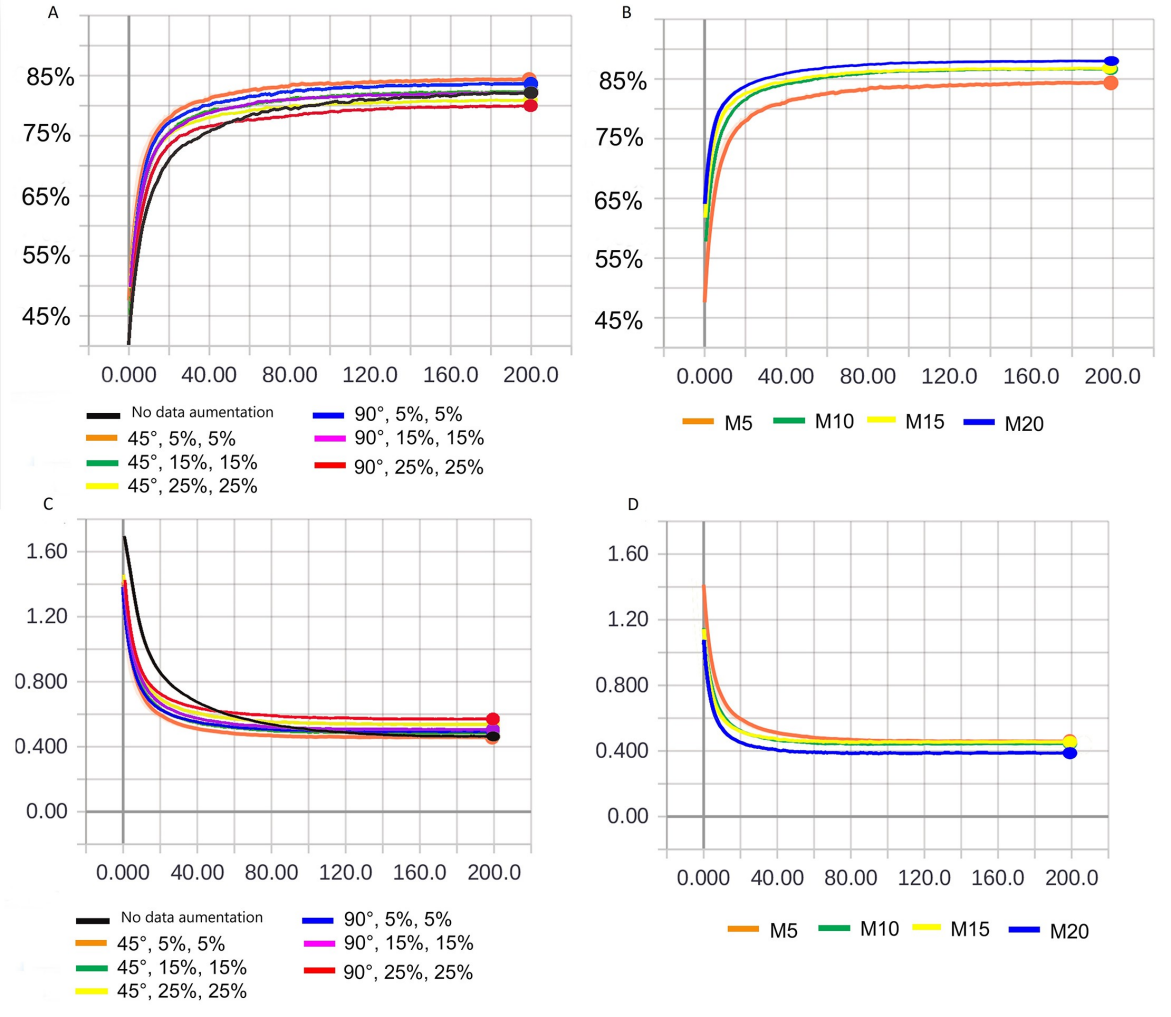
Effect of hyperparameter variation on the accuracy and loss function–data augmentation. (A) The first stage of grid-search plan—accuracy. (B) The second stage of grid-search plan–accuracy. (C) The first stage of grid-search plan–loss function. (D) The second stage of grid-search plan–loss function.

In Fig 7, we depict the behavior of the classification accuracy and cross-entropy function in the validation phases of the experiments for both the stages of hyperparameter optimization to generate new images.

In the first optimization stage (see Fig 7A and 7C), regarding the performance of the evaluated sets, the best results for accuracy and loss function were determined to be 45° of image rotation, 5% of image-width displacement, and 5% of height-shift of images. Because of this performance, this set of values was chosen for data augmentation in the second stage.

In the second optimization stage (see Fig 7B and 7D), the best performance regarding the classification accuracy and cross-entropy function was obtained with the multiplication factor of 20, thereby reinforcing once again that data augmentation enhances the model performance in classification tasks [60].

After performing the hyperparameter-optimization process for data augmentation, the DenseNet201 network obtained 87.7% accuracy in the validation phase after 200 epochs (see Fig 7B, M20). As previously mentioned, the final performance evaluation of the model was performed during the test phase, to verify its generalization capacity. In Table 8, we present the result, which is in the form of a confusion matrix, of the prediction of the model in the test phase, obtained after the second stage of the optimization process.

**Table 8:** Confusion matrix of the model after the optimization process of data-augmentation hyperparameters.

Class	*Ae*. *aegypti* female	*Ae*. *aegypti* male	*Ae*. *albop*. female	*Ae*. *albop*. male	*C*. *quinq*. female	*C*. *quinq*. male	TOTAL	TPR (%)	TNR (%)	BA (%)
***Ae*. *aegypti* female**	201	9	22	3	3	0	238	84.5	95.7	90.1
***Ae*. *aegypti* male**	16	240	20	32	4	0	312	76.9	96.7	86.8
***Ae*. *albop*. female**	24	4	251	9	0	1	289	86.9	94.3	90.6
***Ae*. *albop*. male**	8	26	23	214	0	1	272	78.7	96.0	87.3
***C*. *quinq*. female**	7	1	3	3	190	1	205	92.7	98.0	95.3
***C*. *quinq*. male**	0	0	1	3	19	171	194	88.1	99.8	94.0
**TOTAL**	256	280	320	264	216	174	**1510**			**90.7**
**PR (%)**	78.5	85.7	78.4	81.1	88.0	98.3				

TPR: True-Positive Rate; TNR: True-Negative Rate; BA: Balanced Accuracy.

The overall BA in the classification, up to this phase of the optimization, was 90.7%. In Table 8, we also present the performance of the model for each of the six classes.

At this stage of hyperparameter optimization, although the overall BA result previously exceeds 90%, it was established that each class should have a true-positive rate above 80%. This goal was established, considering the result previously achieved using CNN GoogLeNet, when the general accuracy of 76.2% was obtained. However, of the six classes evaluated, three presented unsatisfactory accuracy (64.3% for the classification of species *Ae aegypti* female, 57.1% for that of *C*. *quinquefasciatus* female, and 71.4% for that of *C*. *quinquefasciatus* male) [41]. Accordingly, it can be seen from Table 8 that for classes *Ae*. *aegypti* Male and *Ae*. *albopictus* male, the model must be improved.

### Optimization of hyperparameters to extract features and for the classification layers

After the optimization of the training and validation dataset-augmentation hyperparameters, the CNN, Xception, and DenseNet201 architectures were investigated. In Table 9, we present the results of the performance of the hyperparameters grid search in terms of features extraction and classification (see Table 6) achieved at the end of the training epochs.

**Table 9:** Grid-search results for the definition of CNN architecture and hyperparameters for the classification layers.

#	CNN	Number of Epochs	Optimizer	LR	Results			
					L_t	Acc_t.	L_v	Acc_v
1	Xception	200	Adam	4e-7	0.76	71.3%	0.75	71.9%
2	Xception	200	Adam	4e-6	0.31	88.8%	0.55	80.4%
3	Xception	200	Adam	2e-5	0.19	93.3%	0.58	81.6%
4	Xception	200	Adam	1e-4	0.08	97.2%	0.70	83.5%
5	Xception	300	Adam	4e-6	0.29	89.6%	0.55	80.9%
6	DenseNet201	200	Adam	4e-6	0.26	90.5%	0.42	85.6%
7	DenseNet201	200	Adam	1e-5	0.18	93.6%	0.41	86.9%
8	DenseNet201	200	Adam	2e-5	0.13	95.3%	0.41	88.0%
9	DenseNet201	200	Adam	3e-5	0.12	95.6%	0.41	88.1%
10	DenseNet201	200	Adam	6e-5	0.08	97.0%	0.41	89.4%
11	DenseNet201	200	Adam	1e-4	0.05	98.1%	0.43	90.0%
12	DenseNet201	200	Adam	2e-4	0.03	99.0%	0.47	90.4%
13	DenseNet201	200	Adam	3e-4	0.02	99.4%	0.51	90.7%
14	DenseNet201	200	Adam	6e-4	0.01	99.7%	0.56	90.8%
15	DenseNet201	200	Adam	1e-3	0.01	99.7%	0.59	90.7%
16	DenseNet201	300	Adam	4e-6	0.23	91.8%	0.41	86.3%

LR: Learning rate; L_t: Training loss; Acc_t: Training accuracy; L_v: Validation loss; Acc_v: Validation accuracy.

From the results presented in Table 9, the five best performances in terms of the accuracy in the validation phase and the five best ones in terms of the loss function in the validation phase were experiments 7, 8, 9, 10, and 11 and experiments 12, 13, 14, 15, and 16, respectively. Therefore, the models corresponding to these experiments were selected for the evaluation of the classification in the test dataset.

**Fig 8:**
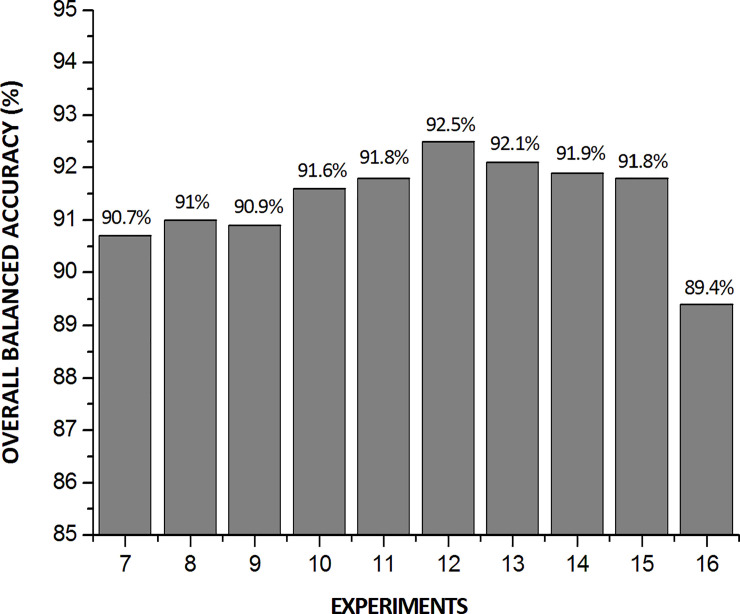
Overall BA of experiments 7–16 of Table 9.

In Fig 8, we depict the results of global BA of experiments 7–16 in classifying *Ae*. *Aegypti* male, *Ae*. *Aegypti* female, *Ae*. *Albopictus* male, *Ae*. *Albopictus* female, *C*. *quinquefasciatus* male, and *C*. *quinquefasciatus* female.

Notably, the values of the initial weights are randomly assigned in a neural network training. As the learning progresses, these weights are adjusted; however, the initial randomness of these values may alter the final performance. The factors that determine the minimum local value are the initial weight and training algorithm. In addition, the weight initialization affects the convergence speed, convergence probability, and generalization [62].

Therefore, the accuracy results depicted in Fig 8 might vary if the models are retrained. To evaluate the effect of this variation on the selection of the hyperparameter set of this study, the two experiments that achieved the best overall accuracy performance (experiments 12 and 13) were retrained twice more, and their new models underwent dataset evaluation again.

**Fig 9:**
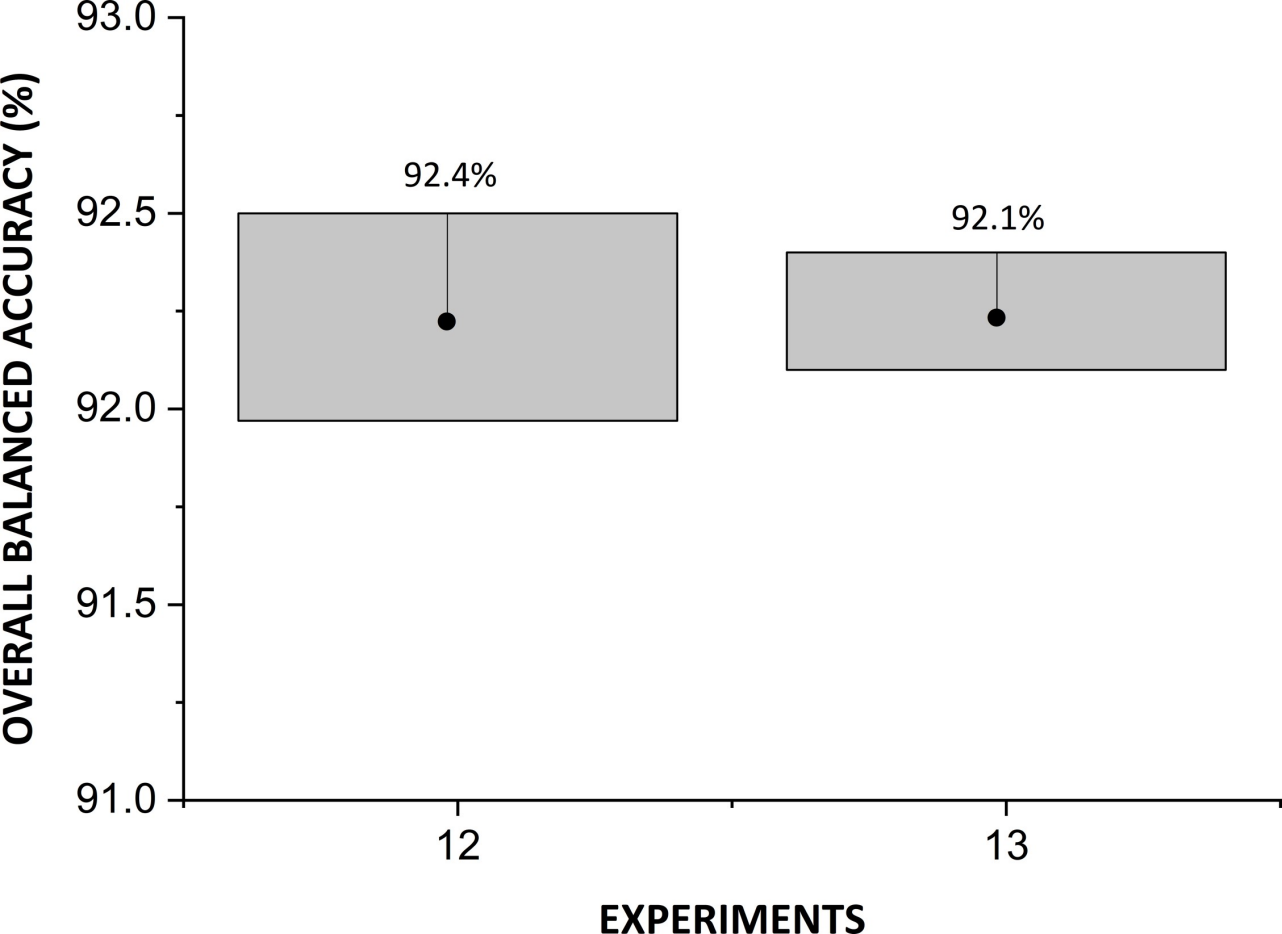
Variation in the global BA of experiments 12 and 13.

In Fig 9, we depict the performances of experiments 12 and 13 (see Table 9) and the variation in the overall accuracy results for three independent trainings.

On the basis of the performance variation, it is concluded that although the model with the hyperparameters of experiment 13 may achieve similar performance to that of the model with the hyperparameters of experiment 12, the latter model, shows superior performance (on average) in terms of the overall BA. Therefore, we selected that model.

**Fig 10:**
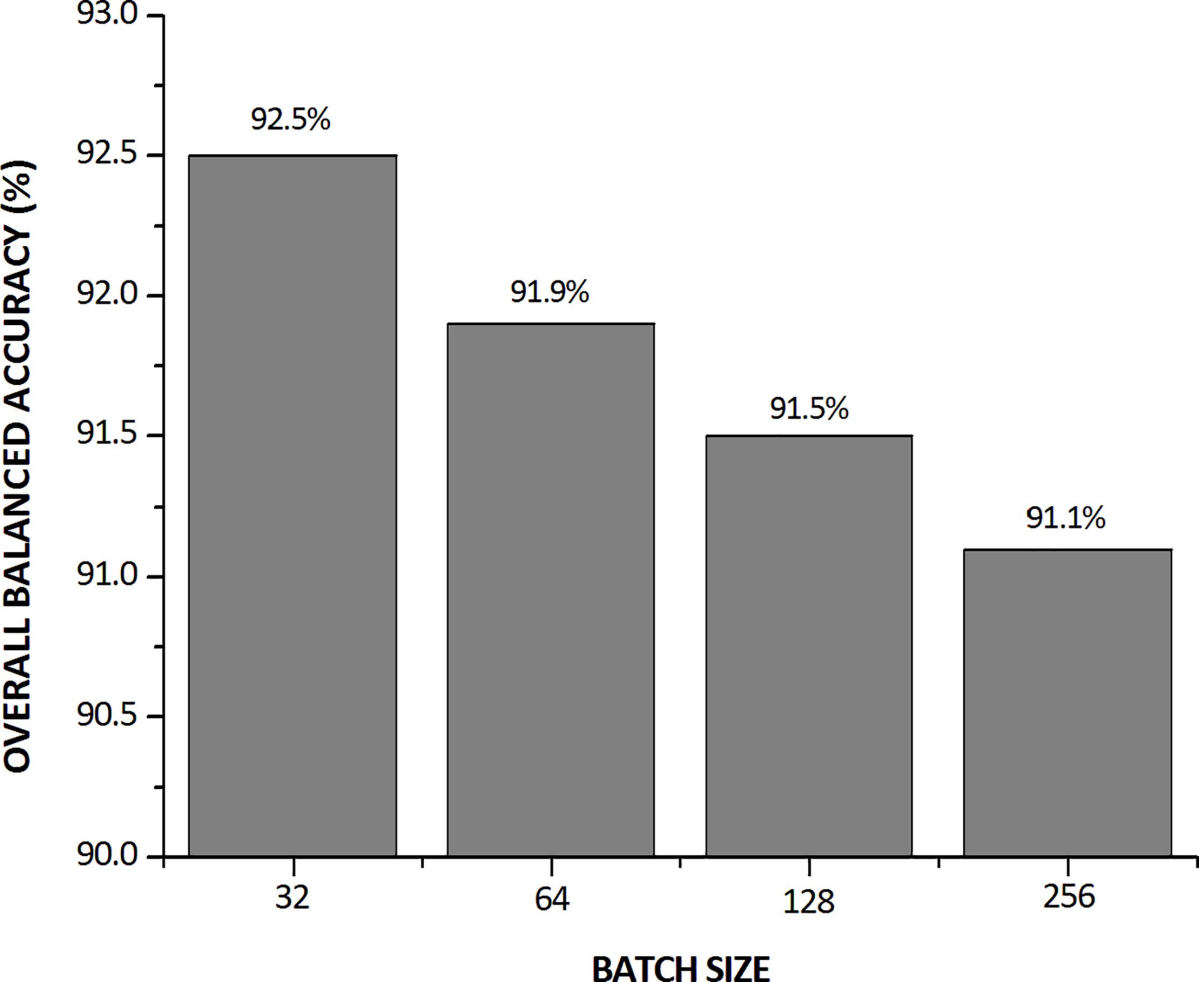
Overall BA upon varying the batch size.

Considering the CNN architecture and hyperparameters of experiment 12 (see Table 9), we began to investigate the batch-size variation. Fig 10 presents the results of the overall BA for various batch sizes.

In the overall result, the batch size of 32 showed the best performance. Therefore, after concluding the entire optimization process, in Table 10 we present the configuration of the hyperparameters that achieved the best performance in terms of the classification of adult mosquitoes in six different classes.

**Table 10:** Configuration of hyperparameters after performing the optimization for the classification of adult mosquitoes.

Hyperparameter	Configuration
CNN	DenseNet201 (pre-trained)
Optimizer	Adam
Number of epochs	200
Learning rate	0.0002
Batch size	32
**Dataset Augmentation**	
Rotation	45%
Width shift range	5%
Height shift range	5%
Multiplier	20
**Reduce Learning Rate on Plateau**	
Monitor	Validation loss (*cross entropy*)
Factor	0.9
Patience	4
Minimum learning rate	0
**Classification Layers**	
*pool0 = GlobalAveragePooling2D()(inputs)*	
*dense0 = Dense(512*, *activation = "tanh")(pool0)*	
*dpo0 = Dropout(0*.*45)(dense0)*	
*dense1 = Dense(64*, *activation = "relu")(dpo0)*	
*dpo1 = Dropout(0*.*35)(dense1)*	
*outputs = Dense(6*, *activation = "softmax")(dpo1)*	

In Table 11, we present the confusion matrix for six classes with the test dataset, after performing training using the hyperparameter configuration listed in Table 10.

**Table 11:** Confusion matrix of the model after the optimization process.

Class	*Ae*. *aegypti* female	*Ae*. *aegypti* male	*Ae*. *albop*. female	*Ae*. *albop*. male	*C*. *quinq*. female	*C*. *quinq*. male	TOTAL	TPR (%)	TNR (%)	BA (%)
***Ae*. *aegypti* female**	216	6	12	3	1	0	238	90.8	96.4	93.6
***Ae*. *aegypti* male**	11	252	18	28	3	0	312	80.8	97.4	89.1
***Ae*. *albop*. Female**	19	4	258	8	0	0	289	89.3	96.1	92.7
***Ae*. *albop*. Male**	8	21	15	227	0	1	272	83.5	96.4	90.0
***C*. *quinq*. Female**	7	0	2	1	194	1	205	94.6	98.2	96.4
***C*. *quinq*. Male**	1	0	1	4	19	169	194	87.1	99.8	93.5
**TOTAL**	262	283	306	271	217	171	**1510**			**92.5**
**PR (%)**	82.4	89.0	84.3	83.8	89.4	98.8				

TPR: True-Positive Rate; TNR: True-Negative Rate; BA: Balanced Accuracy.

Comparing the results of Table 11 with those of Table 8, it is verified that the improvement in the overall accuracy was approximately 1.8%, reaching 92.5%. Notably, with the exception of the accuracy for the classification of *C*. *quinquefasciatus* male, all the other metrics showed improved performances after the optimization process. In addition, for all the classes, the value of the true-positive rate was higher than 80%.

The comparisons presented in this study are references and not absolute, once the authors have used different datasets. Except the comparison with [41].

The application of the model for the six classes can be compared with the results of other authors. Ouyang et al. [37] applied wing beat frequency profiling techniques and evaluated the classification of the same target species as those used in this study. Consequently, they obtained the average accuracy of 79.5% upon using neural networks. Motta et al. [41] used a CNN-based method for image classification and obtained the accuracy of 76.2% upon using the GoogLeNet network.

Comparing the results for each class, in the work of Motta et al. [41], the classes *Ae*. *aegypti* female, *C*. *quinquefasciatus* female, and *C*. *quinquefasciatus* male presented the accuracy of 64.3%, 57.1%, and 71.4%, respectively, whereas in the present study the same classes showed BAs of 93.6%, 93.5%, and 96.4%, respectively.

Notably, however, in the studies by Ouyang et al. [37] and Motta et al. [41], the test database was significantly small, and the accuracy calculation did not consider the dataset unbalance. In addition, among all the classes studied, the value of the true-positive rate was higher than 80%, which is a goal established in this study.

### Performance for two classes: Detection of *Aedes*

As previously shown, the performance of the model was also trained for differentiating *Aedes* from *Culex* with the objective of evaluating the model performance in the detection of the genus *Aedes*.

**Fig 11:**
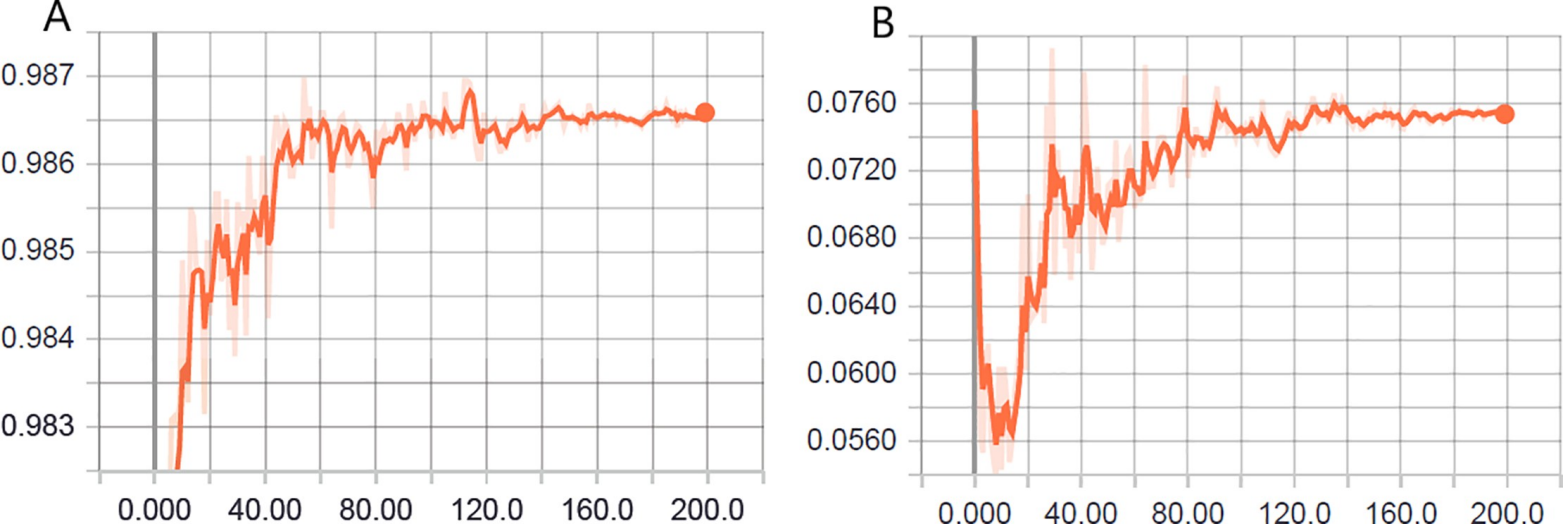
Learning evolution of the model optimized for two classes: (A) accuracy in the validation phase and (B) loss (cross entropy) in the validation phase.

In Fig 11, we depict the learning curve for the optimized-hyperparameter model (see Table 10) for the automatic classification of adult mosquitoes for differentiation between the two classes.

As previously mentioned, the effect of increasing loss function and simultaneous increase in the accuracy should not be confused with overfitting. In addition, it is relevant to consider the scale of the graphs (see Fig 11A and 11B). Both the range of accuracy and loss function are fairly small.

In Table 12, we present the confusion matrix for two classes with the test dataset and, below the Table 12, the calculations of the performance metrics of the 2 x 2 confusion matrix are presented.

**Table 12:** Confusion matrix of the optimized model for *Aedes* detection.

Class	*Aedes*	*Culex*	TOTAL
***Aedes***	1104	7	1111
***Culex***	19	380	399
**TOTAL**	1123	387	**1510**

Precision: $PR=\frac{TP}{TP+FP}=\frac{1104}{1104+19}=98.3\%$

True-Positive Rate: $TPR=\frac{TP}{TP+FN}=\frac{1104}{1104+7}=99.4\%$

True-Negative Rate: $TNR=\frac{TN}{TN+FP}=\frac{380}{380+19}=95.2\%$

Balanced Accuracy: *BA* = 0,5×(*TPR*+*TNR*) = 0,5×(99.4+95.2) = 97.3%

In this stage, the results showed the BA of 97.3% in the detection of mosquitoes of the genus *Aedes*, compared with the mosquitoes of the genus *Culex*. The application of the model, for two classes, can be compared with the results obtained by Sanches-Ortiz et al. [38] and by Reyes et al. [63] for the detection of the *Aedes* mosquito. The first work, despite evaluating the classification in the larval stage and presenting a significantly small number in the dataset, obtained a true-positive rate of 85%. The second work used the SVM technique and obtained the true-positive rate of 92.5%.

Considering the true-positive result of this study, i.e., 99.4%, for two classes with the objective of detecting the mosquitoes of the genus *Aedes*, the proposed model presented an outstanding result.

### Performance for seven classes: Differentiating non-mosquitoes

The model performance was also evaluated for differentiation among seven classes with the inclusion of other insects (spiders, beetles, and bees). The objective in this stage was to evaluate the ability of the model to distinguish mosquitoes from other insects.

**Fig 12:**
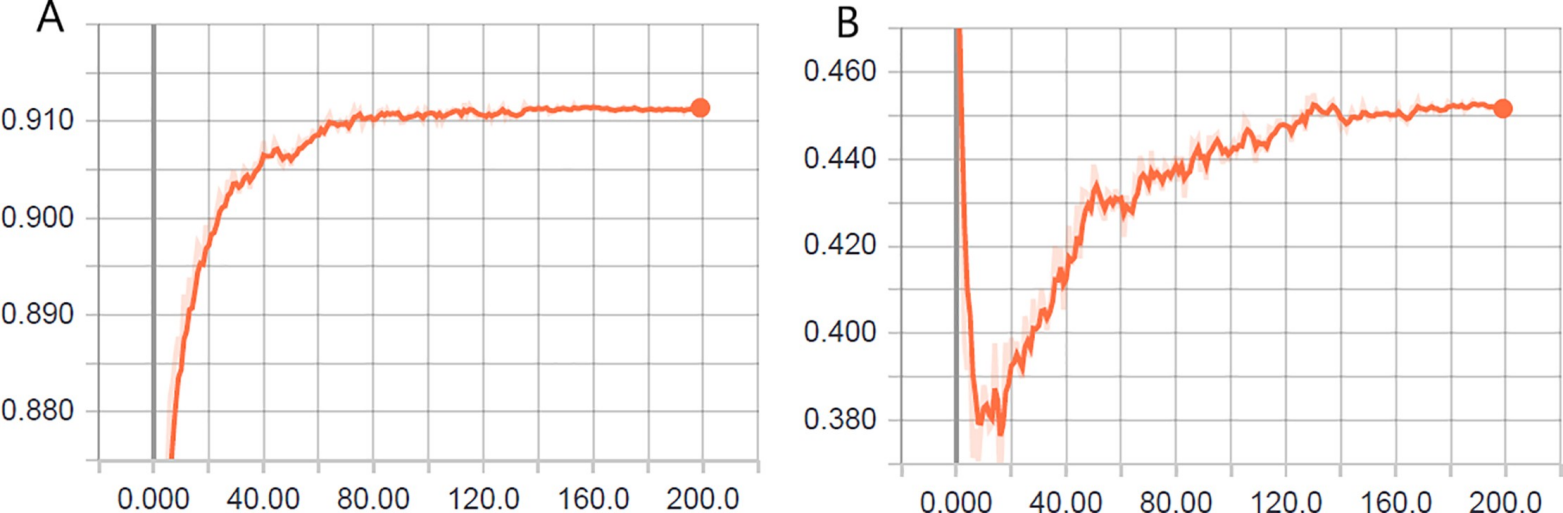
Learning evolution of the model optimized for seven classes: (A) accuracy in the validation phase and (B) loss (cross entropy) in the validation phase.

In Fig 12, we depict the results obtained for the learning evolution of the hyperparameters-optimized model (see Table 10) for the differentiation among seven classes.

The accuracy (91.1%) in the validation phase at the end of 200 epochs for seven classes was higher than the validation accuracy (90.4%) for six classes. These results reinforce the ability of the model to recognize a non-mosquito.

In Table 9, we present the confusion matrix for seven classes with the test dataset. Therefore, the ability of the developed model to distinguish mosquitoes from other insects was observed. With the BA of 98.9%, the “other” class is the one with the best performance compared with the other classes. The overall accuracy at this stage was 93.5% and, expectedly, the true-positive rate was higher than 80% in all the classes evaluated (Table 13).

**Table 13:** Confusion matrix of the optimized model for distinguishing non-mosquitoes.

Class	*Ae*. *aegypti* female	*Ae*. *aegypti* male	*Ae*. *albop*. female	*Ae*. *albop*. male	*C*. *quinq*. female	*C*. *quinq*. male	Others	TOTAL	TPR (%)	TNR (%)	BA (%)
***Ae*. *aegypti* female**	203	9	18	5	3	0	0	238	85.3	97.2	91.3
***Ae*. *aegypti* male**	12	253	9	32	5	0	1	312	81.1	98.1	89.6
***Ae*. *albop*. female**	14	1	260	11	0	0	3	289	90.0	96.5	93.2
***Ae*. *albop*. male**	6	17	19	229	0	1	0	272	84.2	96.7	90.4
***C*. *quinq*. female**	7	0	3	0	192	2	1	205	93.7	98.5	96.1
***C*. *quinq*. male**	0	0	1	1	15	175	2	194	90.2	99.8	95.0
**Others**	3	0	1	0	0	0	233	237	98.3	99.5	98.9
**TOTAL**	245	280	311	278	215	178	240	**1747**			**93.5**
**PR (%)**	82.9	90.4	83.6	82.4	89.3	98.3	97.1				

TPR: True-Positive Rate; TNR: True-Negative Rate; BA: Balanced Accuracy.

The application of the model for seven classes can also be compared with both the results obtained by Ouyang et al. [37], who evaluated the classification of the same target species as those used in this study and obtained average accuracy of 79.5% by using neural networks, and the results obtained by Motta et al. [41], who used the CNN-based method for image classification and obtained the accuracy of 76.2% by using the GoogLeNet network.

### Angle of the mosquito in the image: Influence evaluation on the performance

**Fig 13:**
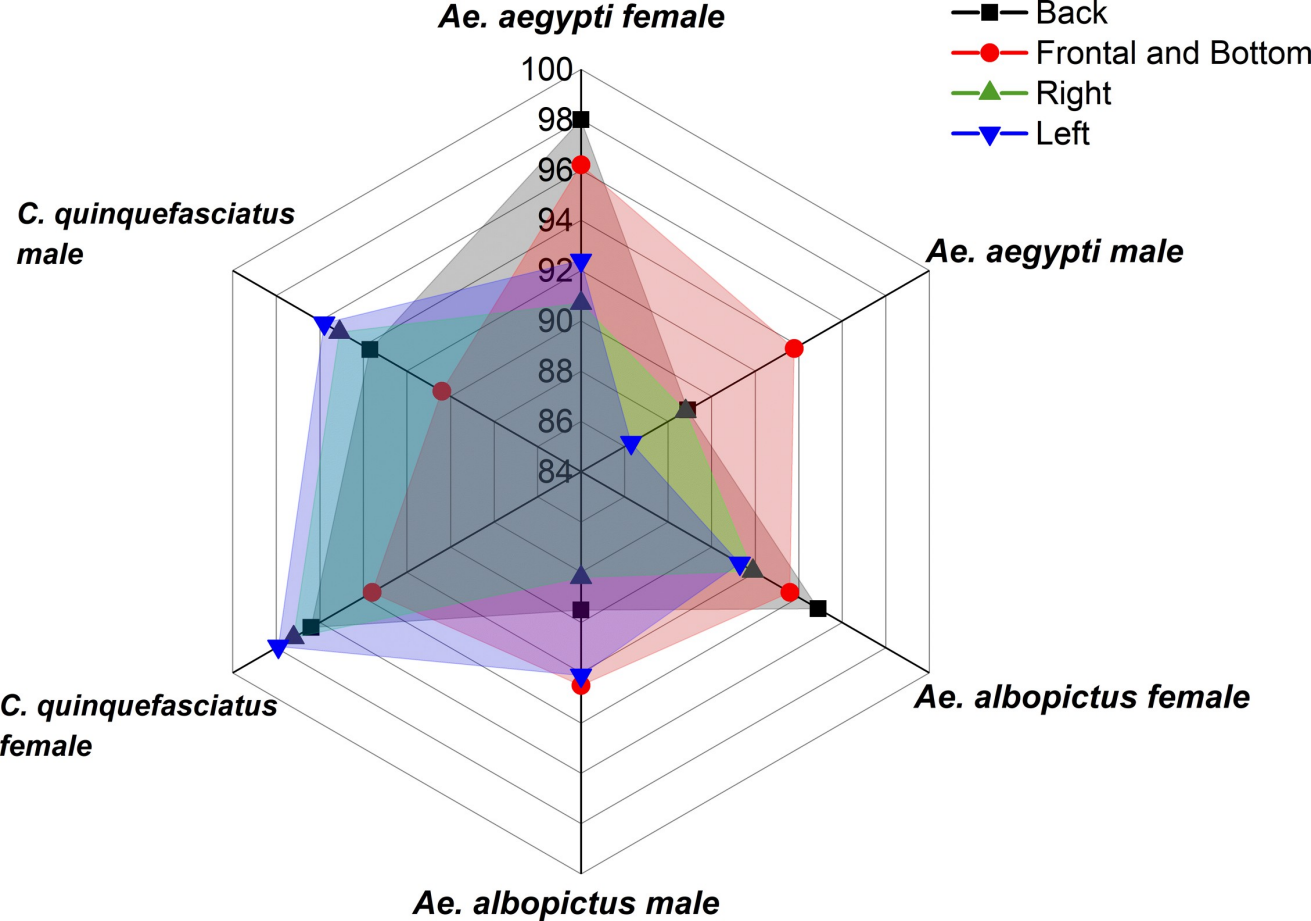
Global and individual BAs for different angles of mosquito in the image.

The optimized model was used to evaluate and classify the images of the test database, and the obtained BA result is presented in Fig 13.

CNN techniques have been used to correct or estimate the orientation of objects in an image. Fischer, Dosovitskiy & Brox [64] focused on estimating and correcting the exact orientation of images and demonstrated that a convolutional network could learn how to predict the canonical orientation of images. Saxena, Driemeyer & Ng [65] proposed a learning algorithm to estimate the 3-D orientation of objects while considering situations with symmetrical and asymmetrical objects.

However, no studies were found that were based on orienting the angle of the object to be photographed in order to acquire an image, for performing automatic classification using CNN.

The analysis of the overall accuracy performance suggests that when the image presents mosquitos on the frontal, bottom, and back angles, the model performs better. However, it is perceived that, depending on the mosquito species, the orientation of the image capture may vary. In the presented case, for the *C*. *quinquefasciatus* species, the captured images of the right and left angles presented high BAs.

## Conclusion

In this study, we showed that using CNN-based models with complex architectures, we could advance the automation of the detection and classification of adult mosquitoes. The performance achieved in the classification and detection of this disease-transmitting vector clearly asserts that a powerful entomological tool, which is based on CNN, can be developed and deployed in the field to control or reduce the economic and social impacts of the arbovirus. In addition, we achieved a significant improvement compared with our previous study, and the improvement is approximately 17% (i.e., from 76.2% in the previous study to 93.5% in this study) in terms of the absolute accuracy.

This tool could help specialists and non-specialists who aim to automatize the classification of species *Ae*. *aegypti*, *Ae*. *albopictus*, and *C*. *quinquefasciatus* and the detection of the genus *Aedes* while achieving high accuracy. It would enormously assist health authorities in controlling vector-borne diseases.

In addition, this study reinforces the need to optimize the hyperparameters of models for a specific application, as a non-automated step. Despite the availability of pre-trained models for object classification, their real-world use requires the prior optimization and evaluation of their performances.

The application of deep-learning techniques requires significant amount of data (hundreds of thousands datapoints). Accordingly, the acquisition of new images is a fundamental step toward developing an entomological tool for field application. The dataset presented in this study is an important step toward building a robust dataset that includes other mosquito species.

By studying the influence of the angle of the mosquito in the image, it is suggested that image capture, should be performed from the front, bottom, or back of the mosquito, especially for the genus *Aedes*. In addition, a specialist must validate the images for their correct labelling. This is essential for the successful application of the model.

Finally, the model with the CNN architecture DenseNet201, Adam optimizer, initial learning rate of 0.0002, and 200 epochs presented the best results for the automatic classification of the adult mosquitoes of species *Ae*. *aegypti*, *Ae*. *albopictus*, and *C*. *quinquefasciatus*. However, new architectures of convolutional networks are continually emerging. Therefore, it is essential that the model for the automatic classification of mosquitoes possesses the flexibility to incorporate new architectures of specific networks or layers that can improve its performance.
